# Update on HIV prevention and management in pregnancy Number 10 – 2025

**DOI:** 10.61622/rbgo/2025FPS10

**Published:** 2025-11-26

**Authors:** Regis Kreitchmann, Geraldo Duarte, Helaine Milanez, Ana Gabriela Travassos, Évelyn Traina, Angélica Espinosa Miranda, Maria Luiza Bezerra Menezes

**Affiliations:** 1 Faculdade de Medicina Universidade Federal de Ciências da Saúde de Porto Alegre Porto Alegre RS Brazil - Faculdade de Medicina , Universidade Federal de Ciências da Saúde de Porto Alegre , Porto Alegre , RS , Brazil .; 2 Faculdade de Medicina de Ribeirão Preto Universidade de São Paulo Ribeirão Preto SP Brazil - Faculdade de Medicina de Ribeirão Preto , Universidade de São Paulo , Ribeirão Preto , SP , Brazil .; 3 Universidade Estadual de Campinas Campinas SP Brazil - Universidade Estadual de Campinas , Campinas , SP , Brazil .; 4 Faculdade de Medicina Universidade do Estado da Bahia Salvador BA Brazil - Faculdade de Medicina , Universidade do Estado da Bahia , Salvador , BA , Brazil .; 5 Universidade Federal de São Paulo São Paulo SP Brazil - Universidade Federal de São Paulo , São Paulo , SP , Brazil .; 6 Faculdade de Medicina Universidade Federal do Espírito Santo Vitória ES Brazil - Faculdade de Medicina , Universidade Federal do Espírito Santo , Vitória , ES , Brazil .; 7 Faculdade de Ciências Médicas Universidade de Pernambuco Recife PE Brazil - Faculdade de Ciências Médicas , Universidade de Pernambuco , Recife , PE , Brazil .

The National Specialized Commission on Infectious Diseases of the Brazilian Federation of Gynecology and Obstetrics Associations (Febrasgo) endorses this document. Content production is based on scientific evidence on the proposed theme, and the results presented contribute to clinical practice.

## Key points

- Pregnancy is the period of greatest risk of infection by the human immunodeficiency virus (HIV) for women, and acute infection during pregnancy has a high potential for transmission of the virus to the baby.
- Combined HIV prevention should be offered to women at risk, including use in pregnant and lactating women, in which Pre-Exposure Prophylaxis (PrEP) and Post-Exposure Prophylaxis (PEP) have proven safe. Pre-Exposure Prophylaxis should be started at least seven days before sexual exposure and can only be discontinued seven days after the last sexual intercourse. Adherence to PrEP (6 to 7 tablets per week) is essential to ensure efficacy in HIV prevention.
- An HIV diagnosis allows access to antiretroviral therapy (ART) that can prevent sexual and perinatal transmission. The partner’s prenatal care includes offering diagnostic tests to the partner and plays a fundamental role in preventing the woman from becoming infected.
- The ART should be started as soon as possible during prenatal care to control viremia and minimize fetal and neonatal exposure to the virus. For women living with HIV (WLHIV), pregnancy represents a period of greater vulnerability to other infections, and the vaccination record should be reviewed and completed during prenatal care, including vaccinations against pneumococcus, influenza, COVID-19, and Tdap.
- The mode of delivery should be defined according to maternal viremia levels in the third trimester. Most women with good adherence to ART will have a viral load (VL) lower than 1,000 copies/mL and may deliver vaginally.
- Breastfeeding always represents a risk of postnatal transmission, and this must be communicated during prenatal care. The distribution of formula together with inhibition of lactation has been recommended by the Brazilian Ministry of Health and this option offers greater safety. Some countries have recently updated their guidelines in this area, including recommendations for counseling, information about risks and support when women wish to breastfeed, provided they have a history of undetectable VL, good adherence to ART and undergo frequent and careful follow-up.
- Safe contraceptive methods, including LARCS (long-acting reversible contraceptives), should be offered to WLHIV for effective reproductive planning.

## Recommendations

- HIV testing should be requested at the first prenatal appointment and repeated in the third trimester and at the time of delivery, as well as in any situation of risk exposure. Rapid tests (RT) are the preferred diagnostic method, as they can expedite the initiation of treatment.
- Pregnant and lactating women who are HIV-negative but have an increased risk of sexual transmission (irregular use of condoms with partners with unknown serology, partners with unsuppressed HIV or unknown VL, sex workers, drug addiction) should be informed about forms of combined prevention, including the use of PrEP. The oral PrEP (tenofovir + emtricitabine) requires the use of additional prevention methods for at least the first seven days of use and quarterly serology testing. PrEP is safe during pregnancy and breastfeeding and prevents transmission, as long as there is good adherence (use of 6 or more pills per week).
- Serodiscordant couples in which the partner is on regular, consistent ART, maintaining undetectable VL, do not present a risk of transmitting the virus. In this situation, there is no need for the HIV-negative pregnant or lactating woman to perform other procedures to prevent vertical transmission, and the use of PrEP is optional and dependent on the woman’s desire.
- The genotyping test is indicated for all pregnant women living with HIV and it is not necessary to wait for the genotyping result to start ART.
- The HIV viral load (HIV-VL) level should be measured at least three times during pregnancy: at the first consultation to establish the magnitude of viremia; two to four weeks after the introduction of ART to assess the response to treatment; and from week 34 to define the mode of delivery.
- ART is indicated for all pregnant women infected with HIV, and the preferred regimen for pregnant women starting treatment is: disoproxil (TDF) + lamivudine (3TC, fixed combined dose, 300/300 mg, once daily) + dolutegravir (DTG, 50 mg once daily).
- For pregnant women who need amniocentesis, the risk and benefit of the procedure and the urgency of its performance must be weighed, taking into account the need for undetectable HIV-VL.
- In cases of postpartum hemorrhage, ergot derivatives should not be administered to patients who have used protease inhibitor antivirals due to the risk of exaggerated vasoconstrictor responses and severe peripheral and central ischemia. Oxytocin or misoprostol should be preferred.
- In cases of premature rupture of membranes (PROM) and/or preterm labor (PTL), expectant management, when less than 34 weeks, does not differ from that adopted for pregnant women not infected with HIV. However, in the case of PTL inhibition, standardized expectant management includes the introduction of maternal intravenous AZT (for those with detectable HIV-VL) concomitantly with drug inhibition of labor, investigation of infectious causes, the use of tocolytics to postpone labor for at least 48 hours, and the use of corticosteroids for fetal lung maturation. Upon successful inhibition of labor, intravenous AZT should be discontinued.
- In women with unknown VL or greater than 1,000 copies/mL, measured after 34 weeks of gestation, elective cesarean section performed from 38 weeks of gestation reduces the risk of HIV vertical transmission (VT). In the others, vaginal delivery is preferred when there is no obstetric contraindication. Every pregnant woman with unsuppressed HIV-VL should receive intravenous AZT during labor or started three hours before elective cesarean section.
- During vaginal delivery, amniotomy should be avoided and AZT infusion should be maintained if HIV-VL is unknown or detectable until the umbilical cord is clamped. The use of medications that increase uterine activity is not contraindicated, provided they follow established safety standards. Delayed umbilical cord clamping (DCC) is recommended by the World Health Organization (WHO).
- In the case of an elective cesarean section, it should be performed from 38 weeks of gestation in order to avoid prematurity, labor and PROM. If the pregnant woman who is indicated for an elective cesarean section starts labor before the scheduled date of surgery and arrives at the maternity ward with minimal cervical dilation (less than 4 cm), the obstetrician should start the intravenous infusion of AZT and perform the cesarean section, if possible, after three hours of infusion; perform complete hemostasis of the abdominal wall, minimizing subsequent contact of the newborn with maternal blood; use prophylactic antibiotics – a single intravenous dose of 2 g of cefazolin.
- During the postpartum period, the patient should be informed about the risks associated with breastfeeding. If the patient agrees, inhibit lactation with the use of cabergoline 0.5 mg, 2 tablets in a single dose immediately after delivery. Upon discharge, reinforce that the newborn should not be breastfed nor should cross-nursing, mixed feeding (human milk and infant formula) and the use of human milk pasteurized at home be permitted. Provide guidance on appropriate use and inform the mother about the right to receive infant formula at least until the newborn is 6 months old. Encourage adherence to ART and accompany the mother in sexual health and reproductive planning actions so that she can make informed and safe choices.

## Background

In 2022, there were 20 million women and 1.5 million children living with the human immunodeficiency virus (HIV) worldwide. ^( 1 )^ The vast majority of pediatric HIV infections result from vertical transmission (VT).

Pregnant women should be advised on the importance of HIV testing during prenatal care and on the benefits of early diagnosis, both for controlling maternal infection and preventing VT. In planned pregnancies with adequate interventions during prenatal care, delivery and breastfeeding, the risk of VT is reduced to less than 2%, ^( 2 )^ whereas without adequate planning and follow-up, it is well established that it ranges from 15% to 45%. ^( 3 )^

To this end, HIV testing of all pregnant women should be performed as soon as possible during pregnancy and if the status of their partners is unknown, they should also be encouraged to test as well. Testing should be repeated in the third trimester and at the beginning of labor for pregnant women with previous negative or unknown tests (strong recommendation). ^( 4 )^

Once diagnosed with HIV, the pregnant woman will undergo an initial clinical and laboratory evaluation, screening for other sexually transmitted infections (STIs), assessment of vaccinations and the need for prophylaxis against opportunistic infections, assessment of situations of social and sexual vulnerability and the need for supportive care, including social, mental health or substance abuse services. A discussion of intrapartum and postpartum considerations, such as use of antiretroviral therapy (ART), mode of delivery, infant feeding, infant antiretroviral (ARV) prophylaxis, timing of infant HIV testing, and family planning/contraception options (strong recommendation) should be included as well. ^( 4 )^

The aim of this FPS from the Brazilian Federation of Gynecology and Obstetrics Associations (Febrasgo) is to guide the management of pregnant and postpartum women and their sexual partners regarding HIV, their potential for VT, the importance of using ARVs and other anti-infectious agents for the prevention and treatment of STIs. It also proposes to review all these steps in a practical, objective and easily accessible way for obstetricians interested in this topic.

### What is the human immunodeficiency virus?

The HIV is a spherical particle measuring 100 to 120 nm in diameter belonging to the genus Lentivirus and the family Retroviridae. Its nucleus has two copies of single-stranded RNA encapsulated by a protein layer or nucleocapsid, capsid and an external envelope composed of a phospholipid bilayer. The HIV genome includes three main genes that encode the structural proteins and viral enzymes: gag, pol and env. The similarity between the genomes of HIV-1 and HIV-2 is approximately 50%. The gag and pol regions of the viral genome show greater similarity between HIV-1 and HIV-2, unlike the env region. Although the HIV-2 and HIV-1 proteins have equivalent functions, they present differences in amino acid composition and molecular weight. ^( 5 )^ HIV classification is performed through phylogenetic analysis of nucleotide sequences of viruses. The current classification is hierarchical and consists of types, groups, subtypes, sub-subtypes and recombinant forms. ^( 6 )^ HIV-1 and HIV-2 are distinct types of the virus and more distant phylogenetically. The genetic variation of HIV has implications for the biology and transmission of the virus, clinical evolution, reactivity and cross-reactions in diagnostic tests that detect the presence of antibodies specific to viral antigens. ^( 5 , 7 )^

### How does HIV act in the individual?

Most HIV-1 infections occur through the mucous membranes of the genital or rectal tract during sexual intercourse. Within the first few hours after sexual infection, HIV and infected cells cross the mucosal barrier, allowing the virus to establish itself at the site of entry and continue to infect CD4+ T lymphocytes, as well as macrophages and dendritic cells. The innate immune response that develops at the site of infection attracts additional T cells, which in turn increases viral replication. From this small population of infected cells, the virus is initially disseminated to local lymph nodes and then, systemically and in sufficient numbers to establish and maintain virus production in lymphoid tissues, in addition to establishing a latent viral reservoir, mainly in memory CD4+ T lymphocytes. ^( 5 )^ Active viral replication and free circulation of the virus in the bloodstream cause the formation of a peak of viremia around 21 to 28 days after exposure to HIV. This viremia is associated with a sharp decline in the number of CD4+ T lymphocytes. Although an immune response is induced in the expansion and systemic dissemination phase, it is late and insufficient in magnitude to eradicate the infection. Immune activation, in turn, produces an additional number of activated CD4+ T lymphocytes that serve as a target for new infections. At the same time, the increasing number of CD8+ T lymphocytes exerts partial control of the infection, although insufficient to prevent (in the absence of therapy) the slow and progressive depletion of CD4+ T lymphocytes and the eventual progression to acquired immunodeficiency syndrome (AIDS). Activation of HIV-specific CD8+ cytotoxic T lymphocytes normally occurs before seroconversion. ^( 5 , 7 , 8 )^ The emergence of an HIV-specific cellular immune response and subsequent synthesis of anti-HIV antibodies leads to a reduction in plasma viral load (VL) (viremia) to an individual-specific set point and to the chronicity of HIV infection. The cell-mediated immune response is more important than the humoral immune response in controlling viral replication during acute HIV infection, but antibodies play an important role in reducing the spread of HIV in the chronic phase of infection. ^( 8 )^

### What are the ways to prevent HIV infection? What is the role of PrEP and PEP in prevention?

There are numerous options for preventing HIV infection and other STIs. The term “combined prevention” refers to the association of several strategies by combining different actions without overlapping one another. These include regular HIV testing, use of Pre-Exposure Prophylaxis (PrEP) and post-exposure prophylaxis (PEP), guidance on safe sex and condom use, diagnosis and treatment of STIs, viral suppression for infected individuals, immunizations and prevention of VT.

Pre-Exposure Prophylaxis consists of the use of oral ARV medications to reduce the risk of HIV infection, and is a safe and effective strategy for people at increased risk of acquiring the disease. ^( 9 )^ The daily use of PrEP provides more than 90% protection against HIV. ^( 10 )^

The PrEP should be considered for people over 15 years of age with a body weight greater than or equal to 35 kg at increased risk of contracting HIV. These are key populations, such as men who have sex with men, transgender people and sex workers, but also other uninfected people who meet criteria according to their sexual practices, number of partners, irregular condom use or any other risk exposure situation. ^( 11 )^

The approach and guidance on prophylaxis should be carried out in a welcoming environment, with attention, respect and free from personal judgments or prejudices, through active and empathetic listening. Candidates for the medication must understand their responsibility for managing their risk of acquiring the infection and assessing their motivation to start prophylaxis.

The literature clearly demonstrates that women without HIV who are exposed to risk situations can benefit from the use of PrEP in reproductive planning, pregnancy and breastfeeding for their children’s and their own protection, considering not only that the risk of acquiring the infection is greater during pregnancy, but also the greater probability of VT in acute infection. ^( 12 , 13 )^ Women with HIV-positive sexual partners, especially those with high or unknown VL, more than one partner with unknown serology, bacterial STI in the previous six months and injectable drug users are considered eligible for the use of PrEP. ^( 11 )^ Health professionals should discuss with sexually active women the indications for PrEP, its benefits and potential adverse effects.

As a previous diagnosis of HIV infection must be ruled out before recommending PrEP, a rapid test (RT) on a sample of whole blood, serum or plasma should be performed. The use of oral fluid is contraindicated and if the RT is not available, laboratory tests can be used.

Testing for syphilis, hepatitis B and C, chlamydia and gonococcus is also recommended whenever available, without the need to wait for the results of these tests to start PrEP. Although the diagnosis of these infections does not contraindicate prophylaxis, the serological profile should be documented in the initial evaluation. Treatment of these diseases and vaccination should be carried out according to routine indications.

Baseline renal function should be assessed by measuring serum creatinine and creatinine clearance (CrCl) and assessing risk factors for renal injury – hypertension and diabetes, use of medications and history of previous renal failure or injury. Previous HIV infection and CrCl below 60 mL/min are contraindications to the use of PrEP. ^( 11 )^

The regimen currently available in the Unified Health System (SUS) is the combination of a fixed dose of tenofovir disoproxil fumarate (TDF) 300 mg and emtricitabine (FTC) 200 mg at a dosage of 1 tablet daily. Eligible individuals may start the medication after testing negative for HIV, preferably on the same day. High concentrations of drugs in mucous membranes are achieved after seven days of medication, but a greater number of weekly doses is necessary for the vaginal mucosa (6 or more tablets per week) than for the anal mucosa (4 or more tablets per week). ^( 14 )^ Therefore, it is essential to advise the user of the importance of maximum adherence to the medication for its effectiveness. The PrEP is started with a double dose on the first day and then, 1 tablet is taken per day, and should only be discontinued 7 days after the last sexual exposure. ^( 11 )^ Follow-up includes assessment of signs and symptoms of acute infection, adverse effects of the medication, adherence, risk exposure, and testing for syphilis and HIV at least quarterly. During pregnancy, consider more frequent testing, depending on the exposure and risk of VT.

Current evidence shows that the use of the TDF+FTC combination as an HIV prevention strategy during pregnancy and breastfeeding is safe for pregnant and breastfeeding women and their babies. ^( 15 )^

Post-Exposure Prophylaxis consists of the use of medication to reduce the risk of infection after potential risk exposure. To decide on the use PEP, it is necessary to assess if the biological material and the type of exposure pose a risk for HIV transmission, if there has been less than 72 hours between the exposure and the start of the medication and if the exposed person is HIV-negative at the time of care. Blood, semen, vaginal fluids, serous fluids, amniotic fluid and cerebrospinal fluid are the biological materials that pose a risk. Risk exposures include percutaneous exposure, mucous membranes, unprotected sexual exposure, exposure to non-intact skin and bite wounds with associated bleeding. The situations that indicate PEP during pregnancy and childbirth are the same as for any other person. A combination of tenofovir and lamivudine (TNF+3TC) 300 mg/day with dolutegravir (DTG) 50 mg/day for 28 days is the medication used. ^( 16 )^

Note that destigmatizing the disease and educating about the risks and possibilities of treatment and prevention are still fundamental points in HIV prevention.

### How to diagnose HIV?

Early diagnosis of HIV helps prevent progression to AIDS, interrupts the chain of transmission and is essential to prevent VT during pregnancy and childbirth.

The most commonly used tests for diagnosing HIV are immunoassays (IA) of the ELISA (Enzyme-Linked Immunosorbent Assay) type. Four generations of IAs have been developed in recent decades, defined according to the evolution of the methodologies used. Third-generation tests simultaneously detect anti-HIV antibodies of the IgG and IgM classes and have a seroconversion window of 20 to 30 days. Fourth-generation tests, in addition to specific antibodies, also detect the P24 antigen, reducing the diagnostic window to approximately 15 days. ^( 17 )^ These tests are performed in a hospital environment and can take up to four hours to be performed. Rapid test are simple IAs performed in the presence of the individual in a non-hospital environment with results in up to 30 minutes. They can be performed on oral fluid and finger prick, in the latter case only in health care settings. Their great advantage is the rapid result and easy access, and are ideal in a series of situations, such as the beginning of prenatal care and admission for delivery and abortion.

The diagnosis of HIV is logically subject to errors that may be due to the immunological window, test limitations, and operational factors. Although rare and of little significance in the context of public health, there are HIV-exposed seronegative individuals (HESN or “immunosilent individuals”) who produce low or absent levels of antibodies, and elite controllers who present undetectable viremia, even in the absence of treatment. ^( 18 )^

Possible causes of false-reactive tests are autoimmune diseases, liver disease, hemodialysis patients, patients who have undergone multiple blood transfusions, recent vaccination against influenza A-H1N1, and pregnancy. Cases in patients with COVID-19 have been described recently. ^( 19 - 21 )^ False non-reactive results may occur in the immunological window in HESN individuals and in people with compromised immune systems. ^( 22 , 23 )^

Prenatal care providers should talk to pregnant women about testing for HIV and other STIs, and HIV testing should be performed at least three times during prenatal care: at the first appointment, at the beginning of the third trimester (around 28 weeks) and at the time of delivery. More frequent testing should be offered whenever there is a risk exposure situation. ^( 4 )^ Remember to approach the sexual partner and offer testing whenever appropriate.

HIV diagnosis should be made by performing two tests, one initial and one complementary. Two or more tests combined form a flowchart with the aim to increase diagnostic sensitivity. Several flowcharts are proposed, ^( 18 )^ and the optimal choice depends on the specific scenario, available resources and testing objective. Performing the RT allows for immediate diagnosis and referral of the pregnant woman, anticipating the timely start of treatment. In the case of a non-reactive RT, the result is released as a “non-reactive sample for HIV”, but it is suggested to complement it with ELISA, since a failure to perform the RT can lead to an extremely harmful diagnostic error during prenatal care. ELISA serology can be used for prenatal diagnosis, but the results and treatment must be timely provided. In the case of a reactive RT, another test is performed at the same time and if the result is confirmed, a complementary test must be requested. The same applies to reactive ELISA serology.

Different methodologies are used in complementary tests, which include the Western Blot (WB), Immunoblot (IB) and molecular tests. The WB and IB are expensive and require subjective interpretation to establish the diagnosis. Molecular tests detect the RNA or proviral DNA and are useful for diagnosis in children under 18 months and in acute infection in adults. Currently, the complementary test indicated for reactive RT or ELISA is the VL. The WB and IB are indicated only if there are disagreements between the serology and the VL, such as in the case of elite control individuals. Once the diagnosis is confirmed, genotyping and CD4+ T lymphocyte count should be requested, and it is not necessary to wait for the results of these tests to start ART.

Correct diagnosis of HIV during pregnancy and childbirth is key to controlling the disease and preventing the VT of an infection that is currently controllable and preventable, but still has no cure.

### What else needs to be done in the prenatal care of HIV-positive pregnant women in addition to the usual prenatal care?

The pregnant woman should be assessed regarding the duration of the disease, history of previous opportunistic diseases, treatments performed and ARV regimens already used, as well as the reasons for changes. A detailed physical examination should be performed to carefully search for clinical signs suggestive of manifestations of the disease, opportunistic infections and other STIs. On the skin: look for seborrheic dermatitis, folliculitis, cutaneous mycosis, molluscum contagiosum or Kaposi’s sarcoma. On the head and neck: look for oral candidiasis and/or hairy leukoplakia in the oropharynx; perform fundoscopy if the CD4+ T lymphocyte count is ≤ 50 cells/mm ^3^ . Check for lymphadenomegaly, hepatomegaly or splenomegaly, and the presence of palpable masses in the abdomen. In the neurological system, check for focal signs and assess cognitive status. Evaluate the lower genital tract by examining the vaginal, anal, and perianal regions, looking for discharge, ulcers, and lesions suggestive of human papillomavirus (HPV) infection or neoplasia. ^( 4 )^

Among laboratory tests, the HIV viral load (HIV-VL) level should be performed at least three times during pregnancy: at the first visit to establish the magnitude of viremia; two to four weeks after the introduction of ART to assess the response to treatment; from 34 weeks onwards to indicate the mode of delivery. The CD4+ T lymphocyte count should be performed at the first visit and at least every three months in pregnant women starting treatment. In those already undergoing clinical follow-up with ART and undetectable HIV-VL, request CD4+ T lymphocyte count at the first consultation and at 34 weeks of pregnancy. Genotyping should be performed together with the first VL and whenever treatment failure is identified. ^( 4 )^

As tuberculosis (TB) is the main defined cause of death in people living with HIV (PLHIV), it must be investigated at all consultations for the presence of respiratory symptoms (cough), fever, weight loss and/or night sweats and probable intimate contact with a person with respiratory symptoms.

The existence of any of these symptoms may indicate active TB and should be investigated. The tuberculin skin test (PT or PPD) should be performed in all asymptomatic women and with no previous history of the disease. If PT < 5 mm, repeat annually, as well as after immunological reconstitution with the use of ART. If PT > 5 mm, treat latent TB infection (LTBI) with isoniazid (INH) 300 mg orally for six to nine months, supplemented with pyridoxine 50 mg daily, provided that active TB has been ruled out. It is recommended that all PLHIV with a CD4+ T lymphocyte count less than or equal to 350 cells/mm ^3^ receive treatment for LTBI after ruling out active TB, regardless of PT. If PPD is unavailable and active TB is ruled out, INH prophylaxis should also be considered for pregnant women in high-burden settings (e.g. prisons or shelters), or those living with individuals confirmed to have TB. For respiratory symptoms (persistent cough ≥ 2 weeks), regardless of the CD4+ T lymphocyte count, a sputum sample should be requested for rapid TB testing (TRM-TB), if available, or two sputum samples for direct screening for Koch’s bacillus by smear microscopy (BAAR) and mycobacterial culture. ^( 4 )^

The laboratory evaluation should include liver function tests (at the initial consultation), including anti-HBs, kidney function tests (repeated quarterly), as well as screening for chlamydia and gonococcus in vaginal secretion (PCR), when available.

As for immunization, the pregnant woman’s previous vaccination status should be checked, and updated if incomplete. Recommended vaccines for these patients are the hepatitis B vaccine, administered in a double dose and added in a fourth dose (for those not previously vaccinated), following the schedule of 0, 30, 60, 180 days, and the COVID-19 vaccine (biannual), meningococcal C conjugate (if not received) and against pneumococcus (every 5 years). ^( 4 , 24 )^

ART is indicated for all pregnant women infected with HIV, regardless of clinical and immunological criteria, and should be maintained after delivery, regardless of the CD4+ T lymphocyte count. ^( 4 )^ Genotyping is indicated for all pregnant women starting ART to guide the choice of the therapeutic regimen with the greatest genetic barrier to resistance associated with greater adherence, aiming at reducing the transmission of HIV strains resistant to one or more classes of ARVs, which determines a greater chance of ART failure. ^( 25 , 26 )^ However, ART may be initiated in pregnant women even before the results of the CD4+ T lymphocyte count, HIV-VL and genotyping tests are available, especially in cases of pregnant women who start prenatal care late, with the aim to achieve viral suppression as quickly as possible. ^( 4 )^ Suppression of HIV-VL is a determining factor in reducing VT. The use of ART during pregnancy reduces the rate of HIV vertical transmission (HIV-VT) from approximately 30% to less than 1%, when maternal HIV-VL suppression (plasma HIV-VL < 50 copies/mL) is achieved close to delivery. ^( 27 )^ Initial therapy should always include combinations of three ARVs, two nucleoside/nucleotide reverse transcriptase inhibitors (NRTIs) associated with a third ARV. Dolutegravir is an ARV from the integrase inhibitor class and is preferred for pregnant women starting ART due to its efficacy in rapidly reducing HIV-VL, absence of teratogenicity, good tolerance, and low drug interaction. ^( 28 , 29 )^ Therefore, the preferred regimen for pregnant women starting treatment should be: TDF + 3TC (fixed combined dose, 300/300 mg, once daily) + DTG (50 mg, once daily). ^( 4 )^ This regimen does not increase birth defects compared to the general population and is very well tolerated during pregnancy. If TDF/3TC cannot be used, the combination of AZT/3TC is the second option. If this combination cannot be used, the use of abacavir (ABC) associated with 3TC is recommended as a third option, but only in those with a NEGATIVE test for HLA-B*5701, due to the risk of hypersensitivity. ^( 4 )^ If it is not possible to use DTG, the alternatives will be the use of raltegravir (RAL) 400 mg every 12 hours, or protease inhibitor (PI) regimens containing ritonavir (r) as a booster (PI/r). Atazanavir (ATV/r) is one of the options, as it has high viral suppression potency and a safety profile during pregnancy, in addition to its convenient dosage (1 300 mg/100 mg tablet once daily). ^( 30 )^ The other option is darunavir (DRV), also with high viral potency, at a dosage of 600 mg associated with 100 mg of ritonavir, twice a day. ^( 3 )^ Efavirenz (EFV) has been used less and less during pregnancy due to concerns about neurotoxicity, in addition to the worldwide increase in the incidence of transmitted resistance to NNRTIs, which occurs when an individual becomes infected with a strain of HIV-1 already resistant to one or more drugs. ^( 31 )^ A survey conducted by the Ministry of Health analyzing the database of pre-treatment genotyping for pregnant women showed an overall rate of mutations that conferred resistance to NNRTIs of approximately 8%, above the 5% cutoff established by the World Health Organization. (WHO). ^( 4 )^ Therefore, the use of EFV must necessarily be conditioned on pre-treatment genotyping. In pregnant women using ART, with a diagnosis prior to pregnancy and VL below 50 copies/mL, it is recommended to maintain the ARV regimen in use. Those who have detectable HIV-VL should be assessed for adherence and will need to undergo genotyping to adapt the ART in use. Among those who start ART or who change the regimen in use, a 1-log drop in HIV-VL collected is expected in 2 to 4 weeks.

### What is prenatal care for the partner and what is it for?

Considering that the space for reproductive decisions and actions related to reproductive events such as pregnancy, childbirth and puerperal period (breastfeeding) are scenarios dominated by women, it became necessary to create an environment that allowed men to truly participate in these decisions. This possibility became more realistic with the creation of prenatal care for the partner, a strategy that embraces, includes and cares for the partner from the perspective of healthy fatherhood and effective collaboration with the partner in all senses. ^( 32 )^

According to the Ministry of Health, ^( 4 )^ prenatal care for the partner can contextualize the importance of the conscious and active involvement of men, adolescents, young adults and older adults in all actions aimed at reproductive planning issues and guarantee the access and embracement of the partner in health services. Well-defined guidelines were published in the Partner’s Prenatal Care Guide for Health Professionals with proposals to expand access and embracement for partners in health services and programs, qualifying health care practices in general within the scope of the SUS. ^( 33 )^

Objectively considering the role of the partner in controlling HIV-VL, there are two strategies. The most effective is to include him in prenatal care on a universal basis. Serological tests (HIV, syphilis, hepatitis B and C), fasting blood glucose, lipid profile and blood pressure measurement are recommended in this care interface. ^( 32 )^ With this principle, the possibility of diagnosing HIV infection, which was previously restricted to pregnant women, then also reaches her partner by adopting this strategy. If she is not infected, it is now perfectly possible to prevent sexual transmission through guidance. This will consequently also prevent the VT of the virus. ^( 34 )^ The second scenario is the participation of the partner in the prenatal care of the pregnant woman who is already infected. This strategy should be encouraged, since it increases the pregnant woman’s adherence to the use of ARVs and sexual practices that pose a risk of other infections. If the partner is not infected, the guidance will be aimed at preventing him from becoming infected with his partner or in other relationships. If he is HIV-positive, the guidance and care will be aimed at maintaining his health. In this scenario, the encouragement of adherence to ARVs is bidirectional, increasing the chances of preventing the VT of the virus. In short, every effort to involve the partner in prenatal care only brings positive results for the health of the family, increasing the chances of healthy parenting. ^( 32 )^

### What are the clinical and obstetric complications and how are they managed?

They are managed in the same way as in pregnant women not infected with HIV, regardless of immunological status. Preeclampsia, HELLP syndrome, hepatic cholestasis and acute liver failure are disorders associated with pregnancy and can be confused with the adverse effects of ARVs, which are usually mild and rarely require interruption or change of the regimen in use.

Nausea and vomiting are common in pregnancy and can make it difficult to take medications. This must be identified and managed in order to ensure good adherence to ART use.

Pregnant women with HIV, regardless of ARV use, have a higher risk of presenting adverse outcomes, such as preterm birth, low birth weight, restricted intrauterine growth and stillbirths, compared to HIV-negative individuals. ^( 35 )^ This indicates the need for careful monitoring of fetal growth and well-being.

In pregnant women with an indication for amniocentesis, the risk and benefit of the procedure should be assessed, taking into account the HIV-VL and the possibility of postponing until it is undetectable, when the risk is significantly reduced. ^( 36 )^ Non-invasive fetal diagnostic methods, when available, should be offered.

The main obstetric complications in pregnant women living with HIV (WLHIV) are highlighted bellow:

#### Postpartum hemorrhage

Ergot derivatives should not be administered to women using cytochrome P, cYp450 and cYp3a4 enzyme inhibitors (PI, in addition to macrolide antibiotics) due to the risk of exaggerated vasoconstrictor responses and severe peripheral and central ischemia. Oxytocin or misoprostol should be preferred.

#### Premature rupture of membranes (PROM) and/or preterm labor (PTL)

When less than 34 weeks, expectant management does not differ from that adopted for pregnant women not infected with HIV. However, in the case of PTL inhibition, expectant management may include the introduction of maternal intravenous AZT for those with detectable HIV-VL, concomitantly with drug inhibition of labor, investigation of infectious causes, use of tocolytics to postpone labor for at least 48 hours, and use of corticosteroids for fetal lung maturation. The pregnant woman should continue to take her own ARVs at the usual times. If labor inhibition is achieved, intravenous AZT should be discontinued. When termination of pregnancy is chosen, the decision regarding the mode of delivery will depend on the condition of the cervix, fetal presentation, presence of uterine activity, and HIV-VL. Knowing that the duration of ruptured membranes is associated with a higher incidence of HIV-VT, in situations of unfavorable cervix, absence of uterine activity or when a prolonged or dystocic labor is expected, a cesarean section should be considered with the intention of avoiding prolonged inductions. If cervical conditions are favorable, the pregnant woman’s HIV-VL is less than 1,000 copies/mL with cephalic presentation, the route of delivery may be vaginal.

## What is the best route of delivery for pregnant WLHIV?

Note that vaginal delivery continues to be the predominant recommendation for the resolution of pregnancy in pregnant WLHIV, as long as the VL is below 1,000 copies/mL after 34 weeks of pregnancy. ^( 31 , 37 )^ Below this count, the probability of VT is low in women using ART and is not influenced by the route of delivery. Cesarean sections have a higher morbidity rate in this population, since the frequency of postoperative complications after cesarean sections (thrombosis, infection, and bleeding) is significantly higher among these women compared to those who undergo vaginal delivery. ^( 38 - 41 )^ Therefore, if there is no obstetric indication, elective cesarean sections are not recommended in pregnant women with HIV-VL below 1,000 copies/mL in order to prevent HIV-VT. ^( 4 )^

In reality, the preparation for childbirth of WLHIV begins in prenatal care, taking care of their health and controlling their VL. This prenatal care is considered highly complex and should be performed in specialized centers, even in conjunction with Primary Health Care units. The immunological health of these pregnant women can be indirectly measured by counting CD4+ T lymphocyte cells, and the risk of HIV-VT is linked to the dynamics of the virus, which is assessed using the VL. The quality of prenatal care is essential to achieve these objectives, as is the pregnant woman’s adherence to ART and its effectiveness, variables that are measured by quantifying the VC. Recent research confirms the fundamental role of ART in controlling HIV-VL and, consequently, the VT. ^( 27 )^

The HIV-VL should be measured at 34 weeks of pregnancy, allowing time for the availability of results to discuss the mode of delivery based on this VL. Maternal VL is the most important laboratory marker of risk for HIV-VT. In pregnant women with VL values above 1,000 copies/mL, elective cesarean section at 38 weeks of pregnancy will be indicated, since it has a clear protective effect against this form of HIV transmission. ^( 4 )^ Some countries, such as Canada and Sweden have guidelines in which cesarean section is indicated with lower VL cutoff values. ^( 42 , 43 )^

In addition to VL ≥ 1,000 copies/mL, cesarean section will also be indicated for pregnant women who did not have their VL assessed during pregnancy or do not have documentation proving the result at the time of delivery. In pregnant women who did not test for HIV during pregnancy, RT for HIV is initially indicated. If the result is positive, the RT should be repeated (different from the first one) or the diagnosis should be confirmed with another technique that allows for a quick result, which depends on each service (ELISA or molecular biology). If these resources are not available to confirm the RT result, the epidemiological situation should be assessed (unsafe sexual relations, history/presence of STIs or use of illicit drugs, among other situations) and the decision about the mode of delivery should be shared with the woman in labor. In most cases, the option ends up being a cesarean section. ^( 31 )^ In practical terms, the definition of the delivery route in WLHIV is the result of both the care provided during prenatal care and the patient’s adherence to measures aimed at controlling HIV-VL and reducing harm to maternal and perinatal health. ^( 44 )^

In summary, four parameters must be considered to translate all the benefits of a cesarean section into efficiency in reducing HIV-VT and avoiding unnecessary cesarean sections and prematurity: ^( 4 , 44 )^

- Maternal VL ≥ 1,000 copies/mL;
- Performed at 38 weeks of pregnancy, confirmed by ultrasound;
- Intact chorioamniotic membranes; and
- Out of labor (or at the beginning of the parturition process, with 2-3 cm of cervical dilation).

## When do we need to use intravenous AZT at the time of delivery?

For pregnant women with HIV and a VL ≥ 50 copies/mL after 34 weeks of pregnancy or poor adherence to the ART regimen, intravenous AZT must be used before the cesarean section and during labor. ^( 31 )^ This indication includes pregnant women without HIV-VL measurement or without supporting documentation, as well as those diagnosed through HIV RT at the end of pregnancy or during hospitalization to terminate the pregnancy. ^( 4 )^

## What precautions should be taken during vaginal delivery?

For WLHIV who opted for vaginal delivery, it is advisable to wait for labor to start spontaneously, following the same criteria used for pregnant women without HIV. When this does not occur, labor induction using the usual pharmacological methods (misoprostol and oxytocin) will be indicated, according to the protocol and safety regimens of each service. ^( 31 )^

If intravenous AZT must be used, the infusion can be done in two ways. With access to an infusion pump, the drug will be administered continuously, at a dose of 2 mg/kg in the first hour, followed by a maintenance dose (1 mg/kg/hour) until the umbilical cord is clamped. Guidelines of the Ministry of Health outline the drip administration according to the woman’s weight. Without access to an infusion pump, AZT should be administered as a 2 mg/kg bolus loading dose, followed by 1 mg/kg hourly maintenance doses. ^( 4 )^

D, discontinuation of the regular ART regimen used by the pregnant woman should be avoided in the peripartum period. In the case of any limitation of the oral route, excess fluid should be avoided during medication intake. ^( 31 )^

In the labor of WLHIV, a welcoming environment and access to private sanitary facilities should be provided, and prolonged fasting should be avoided (allowing light meals and oral hydration on demand). The use of a partogram and systematic assessment of fetal well-being using noninvasive methods are recommended for all of these pregnancies, avoiding amniocentesis, cordocentesis, early amniotomy or tissue assessment of fetal blood pH. ^( 45 )^ Vaginal examinations should be kept to a minimum, and chorioamniorrhexis should be postponed as long as the obstetric situation allows, since prolonged rupture of the membranes appears to increase the risk of HIV-VT. ^( 4 )^ It is important to remember that labor analgesia may be performed in these parturient women.

Instrumental delivery should be avoided, but when indicated, forceps should be preferred over vacuum extraction. ^( 4 )^ Although episiotomy should be avoided in principle, there may be an unscheduled need for this obstetric resource in some situations. In these cases, an assistant should protect the incision using compresses moistened with the same degerming agent used to degerm the birth canal. If there is a need to increase uterine activity in number/intensity, the drugs regularly used for this purpose can be prescribed following the usual safety standards. ^( 45 )^

One of the most controversial topics in obstetric care for WLHIV is the timing of umbilical cord clamping, whether early (EC) or late (LC). However, there seems to be a consensus that umbilical cord milking should not be indicated. Although the WHO has recommended cord clamping since 2014, ^( 46 )^ mainly citing the hematimetric benefits for the newborn, the lack of studies considering the risk of HIV-VT has raised doubts about the adoption of this guideline. Although the beneficial effects of cord clamping on neonatal hematimetry are surely irrefutable, the limited research on HIV-VT cannot provide the obstetrician with confidence to follow this recommendation according to protocol in all cases. ^( 47 )^ In short, the justifications for cord clamping are based more on the beneficial effects of LC than on case studies addressing the risk of this measure for the occurrence of HIV-VT. In 2019, Pogliani et al. ^( 48 )^ published a case series of 32 newborns of mothers with undetectable VL who underwent LC, comparing them with a group undergoing EC. Newborns in the LC group had better hematometric parameters than children in the EC group, and there was no HIV-VT in either group. The caveats about this research are: 1) limited case series and 2) testing only in WLHIV with VL under control. Perhaps the release of LT may be eventually considered in pregnant woman with a sustained undetectable VL and a maternal history of irrefutable adherence. In practice, to date, the guidelines of the Ministry of Health have been followed, which recommends the collection of umbilical cord blood. ^( 4 )^

Basic and universal precautions include the use of personal protective equipment (gloves, mask, goggles, gown and apron), which should be adopted by all health professionals in situations where there is a risk of contact with blood, secretions, excretions with mucous membranes or non-intact skin. In the event of exposure to biological materials (occupational accidents), the exposed person should be quickly assessed to determine the need for PEP. ^( 4 )^

## What are the considerations for cesarean section management?

If an HIV-positive pregnant woman is indicated for an elective cesarean section, care should be taken to confirm the gestational age and schedule the termination of the pregnancy at 38 weeks. Use obstetric parameters (date of last menstrual period, uterine height and early ultrasound, preferably in the first trimester) to do this, as these precautions help to avoid iatrogenic prematurity and reduce the possibility of labor and PROM beginning before the scheduled cesarean section date. ^( 44 )^

If a cesarean section was indicated based on the VL, the use of intravenous AZT will automatically be indicated, which can be administered as a continuous drip (infusion pump) or as a bolus. Consider the first dose as a loading dose (2 mg/kg) in the first hour, followed by two more doses (1 mg/kg), totaling three hours of prophylaxis. ^( 31 )^ In women on ART, avoid its discontinuation, maintain oral use at the usual times, and avoid excess fluid during medication intake. ^( 4 )^

In the event of labor and/or PROM beginning before the scheduled cesarean section date, the pregnant woman should be instructed to quickly seek the hospital unit scheduled for the resolution of the pregnancy. Rapid admission to the hospital will also allow rapid initiation of prophylactic intravenous AZT at the doses already mentioned.

Before performing the hysterotomy, careful hemostasis of the abdominal wall and replacement of contaminated compresses or secondary fields should be ensured to minimize the newborn’s subsequent contact with maternal blood. Whenever possible, remove the fetus while maintaining the chorioamniotic membranes intact. Use antibiotic prophylaxis with cefazolin in a single dose of 2 g intravenously. ^( 4 )^

## Does the postpartum period require any special care?

The postpartum period is extremely important in the care of WLHIV. The focus is still on reducing VT, but also on the continuity of care for people living with a chronic disease who need to maintain their ART and receive adequate guidance regarding contraception and breastfeeding.

During this critical time in the maternal and child care line, issues that may impact ART should be assessed, such as the appropriate use of drug prophylaxis for newborns, the effectiveness of measures to inhibit lactation and adaptation to the use of milk formula.

The obstetric follow-up of women with HIV in the postpartum period is the same as for any other woman, except for situations of complications occurring during childbirth and the immediate postpartum period. A return visit should be scheduled between the 5 ^th^ and 8 ^th^ day and at the 42 ^nd^ day postpartum. Remember that ergot derivatives should be avoided in users of protease inhibitors (they are potent inhibitors of the CYP3A4 enzyme) due to the association with acute ischemia of the lower extremities. ^( 49 )^

When uterine atony results in excessive postpartum bleeding in people receiving protease inhibitors, rectal misoprostol is preferred. ^( 31 )^

Consider that women who do not breastfeed tend to have a shorter period of amenorrhea, and may resume ovulation as early as four weeks after delivery. Therefore, contraceptive guidance should be scheduled for a period of approximately 30 days after delivery, reinforcing the need for sexual abstinence until contraceptive guidance is provided.

## What about breastfeeding? What is the risk involved?

The HIV virus and cells infected by it are secreted in colostrum and breast milk and can lead to VT. Although ARV treatment during breastfeeding reduces the risk of VT during this period, we know it does not eliminate it completely.

Approximately 60%-85% of children born to mothers with HIV will not be infected even without receiving any intervention. Furthermore, 85%-90% of newborns exposed to breastfeeding also do not become infected. These findings require a comprehensive assessment of all factors involved in VT, given the growing understanding of viral dynamics and VT.

Studies conducted in different locations and social contexts demonstrate that breastfeeding in situations where there is no other intervention to reduce VT determines a 9%-16% rate of VT.

Postnatal HIV transmission is strongly associated with breastfeeding, with most transmissions diagnosed during this period estimated to result from it. Although individuals using ART without viral detection have no risk of sexual transmission, this does not yet apply to breastfeeding in relation to VT. In a systematic review of six studies of pregnant women using ART in places where breastfeeding is permitted, postnatal HIV transmission rates of 1.08% at 6 months of breastfeeding and 2.93% at 12 months have been found. ^( 50 )^

The PROMISE (Promoting Maternal-Infant Survival Everywhere) study found postnatal transmission rates of 0.3% at 6 months and 0.6% at 12 months in women who were breastfeeding and using ART appropriately. ^( 51 )^

The reason why undetectable VL does not correspond to zero risk of VT during breastfeeding has not yet been elucidated. Probably, there are cells associated with viral DNA that are not targeted by ART. Even in individuals with viral suppression, these cells associated with HIV DNA can be transmitted and reactivate RNA production, leading to transmission. Furthermore, the presence of mastitis can reactivate viral replication, leading to the cellular activation of these cells with DNA, even in individuals with RNA suppression.

In addition to these factors, adherence to ART in the postpartum period is known to decrease, which increases the risk of transmission.

Alternatives to breast milk are available in places with better economic conditions, although this is not the case in less favored places with limited access to clean water, adequate nutrition and health care. Breastfeeding has been encouraged in these places, along with reinforcement of adherence to ART throughout the period for the postpartum woman, as well as in relation to the use of prophylaxis for the newborn.

On the other hand, in some industrialized countries with better Human Development Indexes (HDI), initiatives are emerging to seek safer alternatives for WLHIV who wish to breastfeed their children. The risks of disclosing the diagnosis, the stigma still associated with the infection, and the well-known benefits of breastfeeding are some of the reasons for the maternal desire to breastfeed. ^( 52 )^ In Canada, Australia, certain European countries and selected services in the United States of America, some initiatives already exist, as long as there is maternal choice for this type of breastfeeding and strict standards are guaranteed, such as sustained undetectable VL throughout pregnancy and proven and guaranteed adherence to ART. ^( 53 - 55 )^

The American, European and British protocols ^( 31 , 56 , 57 )^ similarly suggest some management strategies regarding breastfeeding in WLHIV:

1. Discuss the risks and benefits of breastfeeding, as well as alternatives to breast milk.
2. Ensure adherence to effective ART and address any barriers to medication access, emphasizing the critical importance of maintaining excellent adherence throughout the breastfeeding period.
3. Provide advice on the importance of exclusive breastfeeding, avoiding partial breastfeeding with concomitant formula.
4. Show the benefits of ARV prophylaxis for the newborn. Ensure that the pediatrician who monitors the child feels safe regarding breastfeeding in WLHIV.
5. Monitor maternal VL monthly or every 2 months to confirm viral suppression.
6. Provide guidance regarding signs of mastitis or infection in the newborn’s oral cavity, as well as diarrhea or vomiting, which should lead to immediate discontinuation of breastfeeding.
7. Test the newborn every 3 months during the breastfeeding period.

Regarding the duration of neonatal prophylaxis, there is no evidence that extending it beyond 28 to 40 days provides any additional benefit in reducing the risk of VT in breastfed infants. The HPTN046 study showed no difference in VT rates when comparing a group of newborns who received additional nevirapine prophylaxis for 6 months with a group who received it for only 28 days. However, some experts continue to recommend an additional period of 1-4 weeks of neonatal prophylaxis.

Therefore, breastfeeding management in WLHIV should involve exhaustive guidance on the risks and benefits of this intervention in relation to HIV-VT, reinforcing there is an increased risk comparing to the situation of breastfeeding suppression with rates around 0.3%-0.6%. ^( 51 )^

The 2022 Brazilian protocol, which covers the various realities of WLHIV in the country, maintains the guidance on breastfeeding suppression in WLHIV and the use of milk formula. We reinforce that in this situation, the administration of medication for breastfeeding suppression should be offered as soon as possible in the postpartum period. The use of cabergoline in a single oral dose of 1 mg is recommended. ^( 4 )^

As additional guidelines, cross-nursing and the use of human milk with home pasteurization should be avoided.

## Which contraceptive methods can be used by WLHIV?

In addition to effectiveness, the benefits and risks of each contraceptive method should be taken into consideration when providing contraceptive guidance. ^( 58 )^

Another important point when prescribing contraceptives is the presence of other morbidities, such as smoking, high blood pressure, dyslipidemia, among others.

The use of spermicides and diaphragms is absolutely contraindicated in these women, due to the risk of micro fissure in the vaginal wall and, consequently, an increased risk of HIV transmission and exposure to other STIs. ^( 2 )^

In these situations of HIV infection and use of ART, according to the WHO Eligibility Criteria, there are no restrictions on the use of other contraceptive methods. ^( 59 )^

Condoms (male and female) remain the only methods that offer dual protection, reducing the transmission of HIV and other STIs, in addition to preventing pregnancy. However, relying solely on condoms for contraceptive planning necessitates consistent and correct use by the couple, as typical use failure rates range from 18% to 21%. Therefore, it is recommended to associate another contraceptive method with condom use, reinforcing dual protection. ^( 60 )^

The strategies that do not depend on the user to maintain their effectiveness are represented by surgical methods and long-acting reversible contraceptives (LARCs), that is, those that provide contraceptive effect for 3 or more years (copper intrauterine device, levonorgestrel-releasing intrauterine device and contraceptive implant). It is possible to understand that such alternatives may be more appropriate for users with low adherence to methods that depend on daily reminders or frequent action, especially in populations at greater risk, such as adolescents and drug users.

All ART regimens can be used concomitantly with contraceptive methods. Note that DTG can be safely co-administered with oral contraceptives, since it does not present drug interactions with hormonal contraceptives, that is, it is not metabolized by the CYP3A4 enzyme. ^( 61 - 63 )^

## Final considerations

Preventing HIV-VT requires the coordination of different interventions in the health care provided to women. These interventions range from HIV prevention through alternatives that include offering PREP to women at high risk of infection, to access to highly effective LARCs and rapid diagnosis provided by rapid tests. User embracement in health services, combat of the stigma of the disease, and the quick institution of ART, ensuring the adherence, are essential for WLHIV. Qualified monitoring with decisions based on the best evidence, always focusing on the safety of the pregnant woman and the baby, on choices regarding the delivery method, on issues related to breastfeeding and on the follow-up of the exposed baby are fundamental to obtain the best results and eliminate vertical transmission of HIV.

**Figure 1:**
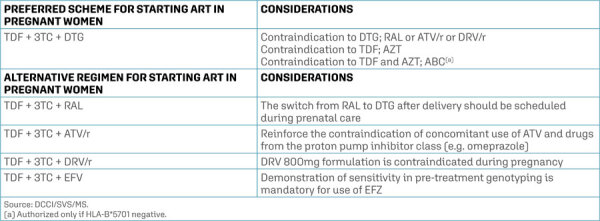
ART regimens for starting treatment in pregnant women with HIV/AIDS

Source: Ministry of Health ^(^^18^^)^
